# Global diversity of the gene encoding the Pfs25 protein—a *Plasmodium falciparum* transmission-blocking vaccine candidate

**DOI:** 10.1186/s13071-021-05078-6

**Published:** 2021-11-08

**Authors:** Pornpawee Sookpongthai, Korawich Utayopas, Thassanai Sitthiyotha, Theerakamol Pengsakul, Morakot Kaewthamasorn, Kittikhun Wangkanont, Pongchai Harnyuttanakorn, Surasak Chunsrivirot, Sittiporn Pattaradilokrat

**Affiliations:** 1 M.Sc. program in Zoology, Faculty of Science, Chulalongkorn University, Bangkok, 10330 Thailand; 2 Department of Biology, Faculty of Science, Chulalongkorn University, Bangkok, 10330 Thailand; 3 Structural and Computational Biology Research Unit, Department of Biochemistry, Faculty of Science, Chulalongkorn University, Bangkok, 10330 Thailand; 4 Faculty of Medical Technology, Prince of Songkla University, Hat Yai, Songkhla, 90110 Thailand; 5 Veterinary Parasitology Research Unit, Department of Pathology, Faculty of Veterinary Science, Chulalongkorn University, Bangkok, 10330 Thailand; 6 Department of Biochemistry, Faculty of Science, Chulalongkorn University, Bangkok, 10330 Thailand

**Keywords:** *Plasmodium falciparum*, Malaria, Vaccine, Transmission-blocking, Diversity, Haplotype

## Abstract

**Background:**

Vaccines against the sexual stages of the malarial parasite *Plasmodium falciparum* are indispensable for controlling malaria and abrogating the spread of drug-resistant parasites. Pfs25, a surface antigen of the sexual stage of *P. falciparum*, is a leading candidate for transmission-blocking vaccine development. While clinical trials have reported that Pfs25-based vaccines are safe and effective in inducing transmission-blocking antibodies, the extent of the genetic diversity of *Pfs25* in malaria endemic populations has rarely been studied. Thus, this study aimed to investigate the global diversity of *Pfs25* in *P. falciparum* populations.

**Methods:**

A database of 307 *Pfs25* sequences of *P. falciparum* was established. Population genetic analyses were performed to evaluate haplotype and nucleotide diversity, analyze haplotypic distribution patterns of *Pfs25* in different geographical populations, and construct a haplotype network. Neutrality tests were conducted to determine evidence of natural selection. Homology models of the *Pfs25* haplotypes were constructed, subjected to molecular dynamics (MD), and analyzed in terms of flexibility and percentages of secondary structures.

**Results:**

The *Pfs25* gene of *P. falciparum* was found to have 11 unique haplotypes. Of these, haplotype 1 (H1) and H2, the major haplotypes, represented 70% and 22% of the population, respectively, and were dominant in Asia, whereas only H1 was dominant in Africa, Central America, and South America. Other haplotypes were rare and region-specific, resulting in unique distribution patterns in different geographical populations. The diversity in *Pfs25* originated from ten single-nucleotide polymorphism (SNP) loci located in the epidermal growth factor (EGF)-like domains and anchor domain. Of these, an SNP at position 392 (GGA/GCA), resulting in amino acid substitution 131 (Gly/Ala), defined the two major haplotypes. The MD results showed that the structures of H1 and H2 variants were relatively similar. Limited polymorphism in *Pfs25* could likely be due to negative selection.

**Conclusions:**

The study successfully established a *Pfs25* sequence database that can become an essential tool for monitoring vaccine efficacy, designing assays for detecting malaria carriers, and conducting epidemiological studies of *P. falciparum*. The discovery of the two major haplotypes, H1 and H2, and their conserved structures suggests that the current Pfs25-based vaccines could be used globally for malaria control.

**Graphical Abstract:**

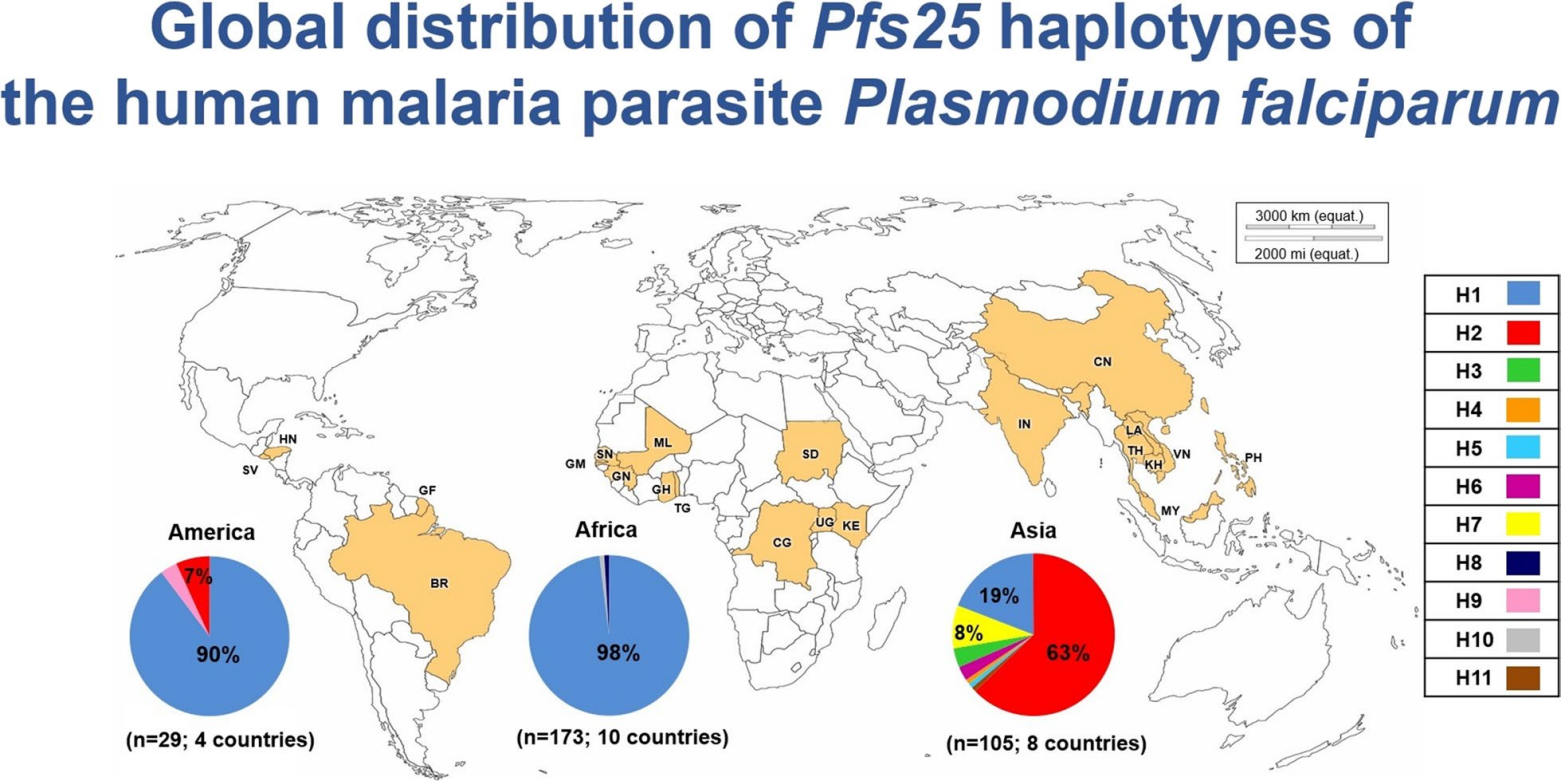

**Supplementary Information:**

The online version contains supplementary material available at 10.1186/s13071-021-05078-6.

## Background

The *Plasmodium* parasite must complete its development in a vertebrate host and transmit to a vector to continue its life cycle. Sexual stages of the malaria parasite, from gametocytes in the vertebrate host to ookinetes in the mosquito, offer potential targets for malaria intervention [1–3]. Although the numbers of gametocytes in the blood circulation are relatively fewer than the blood-stage parasites, and gametocytes can weakly induce immune responses [4], studies have shown that antibodies induced by vaccination with gametocyte and gamete antigens, such as Pfs48/45, Pfs47, and Pfs230, could interfere with gametocytogenesis and thereby may reduce the number of gametes and/or block fertilization [5–7]. Similarly, zygote- or midgut ookinete-targeting antibodies, such as Pfs25, have been experimentally induced and shown to effectively block malaria transmission [8–10]. These antigens are the leading candidates for transmission-blocking vaccine development. Such vaccines aim to reduce the spread of the malaria parasite among humans by preventing infections by *Anopheles* mosquito vectors, thereby representing an important tool for malaria control [11].

Pfs25, a Cys-rich protein comprised of 217 amino acid residues, has a molecular weight of 25 kDa [12] and is encoded by the *Pfs25* gene that spans 654 bp in size and is located on chromosome 10 of *P. falciparum* [13, 14]. Pfs25 expression, which can be detected as early as in macrogametes, dramatically increases in the zygotes and maturing ookinetes [15, 16]. Pfs25 contains a predicted signal sequence at the N-terminus, followed by four tandem epidermal growth factor (EGF)-like domains attached to the anchor domain at the C-terminus; the structure is stabilized with 22 Cys forming 11 disulfide bonds [17]. The EGF-like domain acts as a ligand interacting with laminin, which is located at the basal lamina of the midgut epithelium of the mosquito. The interaction between Pfs25 and the laminin proteins forms the receptor/ligand complex, which can regulate the development of the malaria parasite in the mosquito vector [18]. Homologs of *Pfs25* have been identified in other species of human malaria parasite *P. vivax* (*Pvs25*) and in a rodent malaria parasite *P. berghei* (*Pbs25*) [19–21]. The genetic disruption of *Pbs25* results in a partial inhibition of malaria transmission to *A. stephensi*, whereas double knockout of *Pbs25* and ookinete gene *Pbs28* results in a nearly 100% reduction in oocyst formation, thereby blocking malaria transmission completely [22]. These studies demonstrate that *Pfs25* could play an important role in the sexual stage development of the malaria parasite. Furthermore, *P. berghei* expressing *Pfs25* is susceptible to anti-Pfs25 antibodies of humans [23, 24]. Thus, this system could provide a platform for assessing and optimizing Pfs25-based vaccines.

Currently, recombinant Pfs25 proteins have been produced in different heterologous expression systems and are the only sexual stage antigen tested in clinical trials [25–29]. The vaccine, Pfs25-B, was originally made from a synthetic antigen containing amino acids 22–190 of the natural 217 amino acid precursor protein of *P. falciparum*, 3D7 (cloned line originally derived from isolate NF54) [12, 25]. The antigen was modified by removing the signal peptide at the N-terminal and the hydrophobic region. Subsequently, a second-generation vaccine, TBV25H (later known as Pfs25H), was constructed by addition of the last Cys residue of the fourth EGF-like domain, mutagenesis of Asn-linked glycosylation sites with Glu rather than Ala, and addition of a 6 His-tag for efficient purification [30]. Subsequently, many modifications have been employed to increase the homogeneity and conformational integrity of the vaccine antigen and to boost immunogenicity [31, 32]. Data from phase 1 clinical trial have shown that the Pfs25 vaccine formulation, Montanide ISA 51 (Pfs25/ISA51), showed good immunogenicity; sera obtained from vaccinated volunteers contained transmission-blocking antibodies [33]. Subsequently, Pfs25 vaccine was formulated in Alhydrogel® and conjugated with a recombinant detoxified ExoProtein A from the common Gram-negative bacterium *Pseudomonas aeruginosa* [29, 34]. Phase 1 trial results showed that the vaccine was safe and well tolerated in healthy adults, supporting their further evaluation in clinical phase 2 trials. More recently, Pfs25 has been incorporated in a multi-stage malaria vaccine, RTS,S and Pfs25-IMX313, which has been tested in a pre-clinical trial [35]. Taken together, these studies support the view that Pfs25 is the leading candidate for malaria transmission-blocking vaccines.

One important aspect of vaccine development against the malaria parasite is the antigenic diversity of the vaccine candidates. Genetic polymorphisms in the malaria parasite, caused by mutations during the parasite development in the mammalian host and vector as well as through sexual recombination, could allow the malarial to escape the immunity of the host [36]. Additionally, this could impede vaccine development since the vaccines can target only a specific subset of antigens circulating in the malaria parasite populations [37]. Genetic analyses of the antigens expressed in the blood stage of the malaria parasite, including merozoite surface proteins and apical membrane antigens, have revealed extensive polymorphisms [38–40]. In contrast, the sexual stage antigens are believed to be less polymorphic, and so analysis of their genetic diversity is often neglected. To date, only a few studies have reported the sequence diversity of the ookinete antigen Pfs25. One study analyzed the promotor sequences of *Pfs25* and identified short deletion mutations in the promotor region, which were associated with the inactivation of promoter activities and reduction of gametocyte production in *P. falciparum* [41]. Two studies have reported full-length *Pfs25* sequences of *P. falciparum*, 10 laboratory strains, and 41 isolates from Burkina Faso and Thailand (from Tak province, Western Thailand) [10, 13]. The studies revealed that there were only two *Pfs25* haplotypes (haplotype 1 and haplotype 2) that differed by one amino acid substitution at position A131G. Other studies have reported partial sequences of *Pfs25* from *P. falciparum* isolates in India [42, 43]. Here, the authors identified the A131G mutation and a novel mutation at V131A (haplotype 3). However, none of these studies have combined *P. falciparum* sequence data from public databases to illustrate the global diversity of *Pfs25* in the endemic population worldwide.

Thus, the objective of this study was to determine the global genetic diversity of *Pfs25*, a transmission-blocking candidate gene. We sequenced *Pfs25* from 83 isolates of *P. falciparum* from malaria hotspots in Thailand and combined them with 224 sequences from public databases to construct a global database of *Pfs25* in *P. falciparum*. The sequence analysis of our findings will provide an important insight into the diversity and evolutionary relationship among the *Pfs25* variants and reveal useful information for vaccine design.

## Methods

### Malaria parasite *P. falciparum* from Thailand

**Fig. 1:**
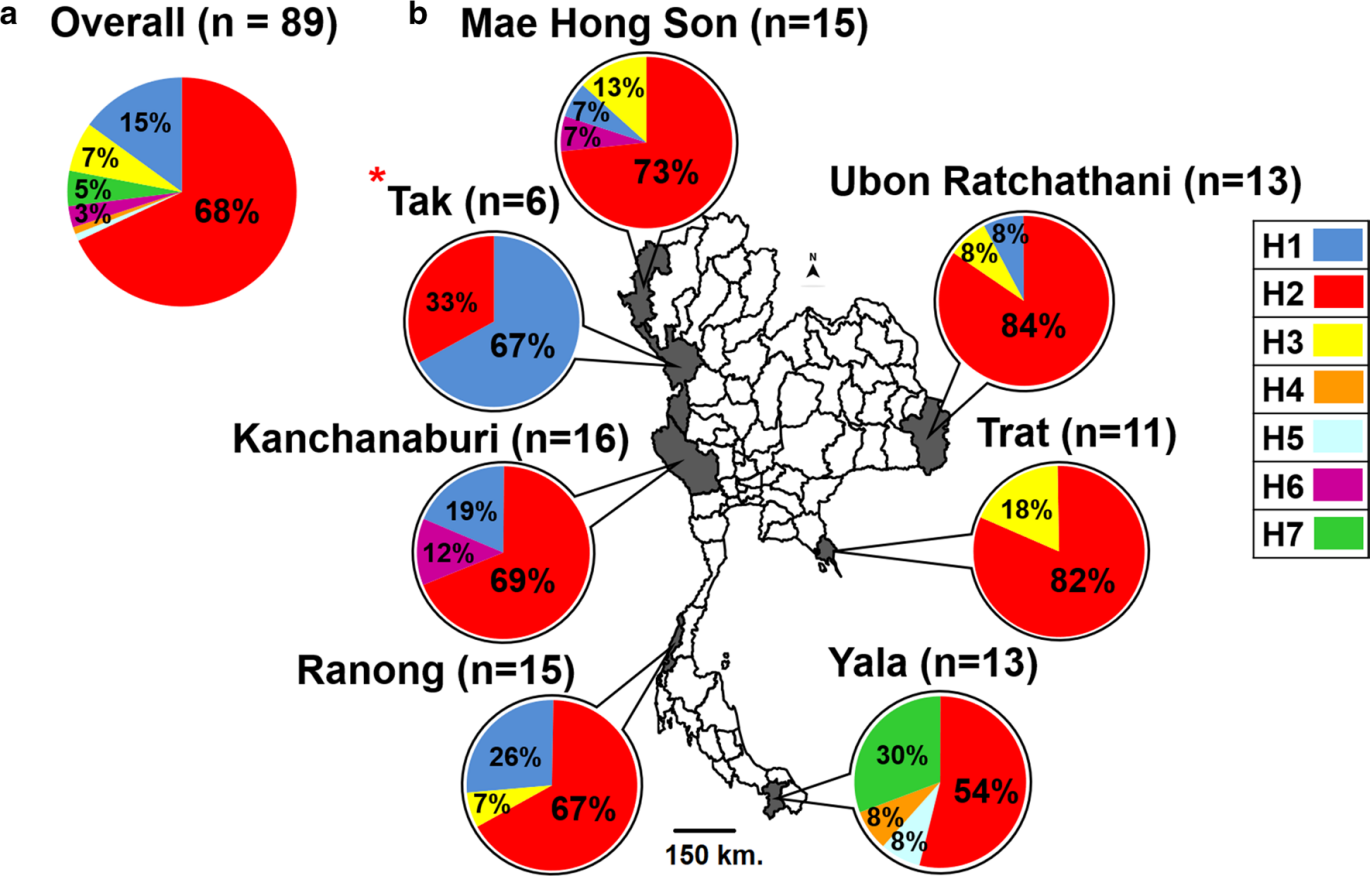
Distribution of *Pfs25* haplotypes in *Plasmodium falciparum* from Thailand. The proportion of *Pfs25* haplotypes in **a** all *P. falciparum* populations in Thailand and **b** in six subpopulations. Four sites (Mae Hong Son, Tak, Kanchaburi, and Ranong) were located along the Thailand-Myanmar border. Trat, Ubol Ratchatani, and Yala were located at the Thailand-Laos, Thailand-Cambodia, and Thailand-Malaysia borders, respectively. *Data were from Da et al. [10]

*Plasmodium falciparum* was isolated from patients from six areas in different regions of Thailand (Fig. 1) from 2001 to 2018. A total of 15, 16, and 15 isolates were obtained from Mae Hong Son, Kanchababuri, and Ranong, respectively, in the northwestern region along the Thailand-Myanmar border. Thirteen isolates were obtained from Ubon Ratchathani located at the Thailand-Laos border. Eleven isolates were obtained from Trat at the Thailand-Cambodia border. Additionally, 13 isolates were obtained from Yala at the southern Thailand-Malaysia border. In total, 83 isolates of *P. falciparum* were included in this study. The malaria parasite was characterized genetically using various markers to ensure that the parasites were clonal [38, 44]. All samples are maintained at Malaria Laboratory, Department of Biology, Faculty of Science, Chulalongkorn University. The parasite pellets were harvested from in vitro cultures and stored at − 80 °C for genomic DNA extraction.

### Generation of *Pfs25* sequences

Genomic DNA was extracted from the parasite pellets using Nucleospin Blood Kit (Qiagen, Thailand) according to the manufacturer’s instructions. The genomic DNA was dissolved in TE buffer and stored at − 20 °C. The genomic DNA was used as the DNA template for amplifying the *Pfs25* sequence using a standard polymerase chain reaction (PCR). Forward and reverse primers used were: 5ʹ-TGTTTTAACCTTGATAATTTACCATTT-3ʹ and 5ʹ-TCTTTGTTTTCTTCAATTTATTCAT-3ʹ, corresponding to the nucleotide positions 1,253,362–1,253,388 and 1,254,216–1,254,240, respectively, of chromosome 10 of *P. falciparum* strain 3D7 (NCBI reference sequence: NC_037281.1 [45]). The PCR reaction mix (20 µl) contained 4 µM forward and reverse primers, 2.5–20 ng/µl DNA template, 0.2 mM dNTPs, 4 mM MgCl_2_, 1 × PCR buffer, and 5 units of Hotstart Taq DNA polymerase (Biotechrabbit, Germany). The PCR conditions were: denaturation at 95 °C for 5 min, followed by 40 cycles of 95 °C for 30 s, 56 °C for 30 s, and 72 °C for 1 min, and then a final 60 °C for 5 min. The PCR products were analyzed using standard agarose gel electrophoresis, and all PCR samples produced a single amplicon. The PCR products were submitted to commercial Sanger DNA sequencing (Bioneer, South Korea). Sequence reactions were conducted using the two PCR primers and one additional sequencing primer, 5ʹ-TTTGTTTCTTTTCCTTTTCATTCA-3ʹ. All the sequences were analyzed in triplicate. After the chromatograms had been manually inspected, the sequences were assembled to generate the full-length coding sequences of *Pfs25*.

### Construction of the global database of *Pfs25*

The *Pfs25* sequences of *P. falciparum* were downloaded from the NCBI and PlasmoDB public databases. To retrieve *Pfs25* sequences from the NCBI website, BLASTn (version 2.10.0) queries were conducted and the reference sequence was set as the *Pfs25* sequence of *P. falciparum* 3D7 (ID: NC_037281.1). The databases for BLASTn search included the nucleotide collection (nr/nt) and whole-genome shotgun contigs. The cutoff was *E* value > 0.1. Additionally, publications reporting *Pfs25* sequences of *P. falciparum* were searched against the PubMed database in the NCBI website. *Pfs25* sequences were also retrieved from PlasmoDB [46]. Partial sequences of *Pfs25*, sequences of the same parasites (same clones or isolates), and sequences with ambiguous nucleotides were excluded from the analysis. The names of the parasite strain or isolate, country, and sequence ID were recorded to generate the global database of *Pfs25* sequences of *P. falciparum*.

### Sequence polymorphism analysis

Full-length coding sequences of *Pfs25* were aligned in molecular evolutionary genetics analysis (MEGA) version 7.0 [47]. Each unique sequence was identified and treated as a haplotype. The genetic diversity indices, including *H* (number of haplotypes), *Hd* (haplotype diversity index), π (average number of nucleotide differences per site between two sequences), and *k* (average pairwise nucleotide difference between the sequences), were calculated using DnaSP version 6.0 [48]. The sliding window plots of π values were plotted against the nucleotide position with a window length of 100 bases and by moving the window in 3-bp steps. The haplotype network was constructed using PopART software (version 1.7) [49]. The median-joining network model was chosen for constructing the haplotype network.

### Evidence of natural selection

Three neutrality tests (Tajima’s *D*, Fu and Li’s D*, and Fu and Li’s F* [50, 51]) were used for detecting the signature of natural selection using the DnaSP 6.0 software. The neutrality tests determine whether a polymorphism occurs at higher or lower frequencies than expected under a neutral model. Additionally, comparison of the mean number of non-synonymous substitutions per non-synonymous site (*d*_*N*_) and synonymous mutations per synonymous site (*d*_*S*_) within each isolate were estimated using Nei and Gojobori’s method, with the Jukes and Cantor correction, as implemented in MEGA 7.0 software [47]. The *d*_*N*_˗*d*_*S*_ values were calculated to investigate the evidence of natural selection, where *d*_*N*_˗*d*_*S*_ values > 0 imply a positive selection (selection favors an excess of non-synonymous mutations over synonymous mutations); *d*_*N*_˗*d*_*S*_ values < 0 imply a purifying selection [52]; and no difference between *d*_*N*_ and *d*_*S*_ (*d*_*N*_ = *d*_*S*_) implies neutral selection. Sliding window plots, with a window length of 100 bases and a step size of 3 bp, were generated for the neutrality tests, and *d*_*N*_˗*d*_*S*_ was used to identify regions of *Pfs25* where a significant departure from neutrality was observed (*P* < 0.05).

### Tests for population differentiation

Distribution patterns (ratios) of *Pfs25* haplotypes of *P. falciparum* in different geographic populations were tested using Wright’s fixation index (*F*_*st*_) analysis in Arlequin version 3.5 [53]. Significant *F*_*st*_ was deemed at *P* < 0.05, indicating population differentiation between parasite population pairs.

### Phylogenetic tree construction

Multiple sequence alignments of *Pfs25* were generated using the MUSCLE algorithm in MEGA 7.0 [47]. The dataset contained 11 unique *Pfs25* nucleotide sequences (haplotypes). A phylogenetic tree analysis was performed using the Bayesian inference (BI) approach implemented in BEAST (version 1.10.4) [54]. Hasegawa-Kishino-Yano + G (HKY + G), combining different equilibrium frequency distributions with unequal transition and transversion rates, was chosen as the substitution model. The posterior probability of the Bayesian tree was calculated by Markov chain Monte Carlo probability distribution sampling algorithm with a chain length of 10,000,000 and log parameters every 1,000. The BI tree was annotated with TreeAnnotator 1.10.4 using a burn-in of 10%. A phylogenetic tree based on the neighbor-joining (NJ) approach was constructed using PAUP [55], using the HKY85 model for NJ tree construction [56]. The reliability of the NJ tree was assessed by the bootstrap method of 1,000 pseudo-replicates. All trees were visualized with FigTree 1.4.3. Sequences of *Prs25* gene from *Plasmodium reichenowi* chromosome 10 (NCBI ID: LT969573.1 and LVLA01000011.1 at nucleotide positions 1,179,907–1,180,560 and 1,168,471–1,169,124, respectively [57]) were used as the outgroup.

### Structural analyses of Pfs25 variants

SWISS-MODEL server [58–62] was employed to construct the homology model of the *Pfs25* haplotype of *P. falciparum* 3D7 (haplotype 1) based on the crystal structure of *Pfs25* in complex with the human transmission-blocking antibody (PDB ID: 6PHB [8]), which has the highest sequence identity to the target sequence and contains the unique amino acid residues of Q91, Q144, and Q166 that are different from H1 and H2. Employing the LEaP module of AMBER18 [63], a model of H2 was constructed from the homology model of H1 by mutating G131 to A131.

All ionizable amino acids of H1, H2, and the *Pfs2* crystal structure were protonated at the physiological pH (pH 7.4) using the H^++^ server [64]. Using the LEaP module of AMBER18 [63] and protein ff14SB force field [65], each system was immersed in an isomeric truncated octahedral box of TIP3P water molecules with a buffer distance of 13 Å and was then minimized to remove unfavorable interactions using a five-step procedure [66–74]. All steps included 2,500 steps of steepest descent and 2,500 steps of conjugate gradient with different restraints on the proteins. Initially, a force constant of 10 kcal/(mol Å^2^) was applied to restrain the heavy atoms of the protein, whereas those of water molecules and hydrogen atoms were minimized. Force constants of 10, 5, and 1 kcal/(mol Å^2^) were subsequently applied to the protein backbone. Finally, the entire system was minimized without any restraining force.

The GPU (CUDA) version of the PMEMD module [75–77] was employed to simulate all systems under the periodic boundary condition. All bonds involving hydrogen atoms were constrained using the SHAKE algorithm [78], allowing 0.002 ps time step simulations. Temperatures of all simulations were controlled with a collision frequency of 1.0 ps^˗1^ using the Langevin dynamics technique [79]. All systems were simulated from 0 K to the physiological temperature of 310 K for 200 ps in the NVT ensemble, while a force constant of 10 kcal/(mol Å^2^) was applied to restrain the backbones of the proteins. The systems were subsequently equilibrated at 310 K for 300 ps with no restraining force in the NVT ensemble. Finally, these systems were subsequently simulated at 310 K and 1 atm for 100 ns in the NPT ensemble.

Employing the cpptraj module [80] of AMBER18, the root mean square deviation (RMSD) values of the simulated structures with respect to the minimized structures were calculated to analyze the stability of the system (Additional file 1: Fig. S1). Since the trajectories in the range of 80 to 100 ns of all systems were found to be stable, based on their RMSD values, these trajectories were used for further analyses. The values of root mean square fluctuation (RMSF) of the backbone atoms of each residue were computed to analyze the structural flexibility. Definition secondary structure of protein (DSSP) was calculated to elucidate the percentages of the secondary structures of all systems. The secondary structures of the Pfs25 variants were also predicted using JPred4: a protein secondary structure prediction server [81].

## Results

### Haplotype analyses of *Pfs25* in Thailand

*Pfs25* sequences of *P. falciparum* from Thailand were retrieved from GenBank and PubMed databases. Six full-length *Pfs25* sequences (654 bp in size) of *P. falciparum* isolates from the Tak province, northwestern Thailand, were obtained [10]. The sequences were classified into two haplotypes: haplotype 1 (*n* = 4, same as that of *P. falciparum* 3D7) and H2 (*n* = 2).

To expand the database, genomic DNA of *P. falciparum* isolates from six other endemic regions in Thailand was obtained (*n* = 83) and used as templates for PCR amplification of the full-length coding sequences of *Pfs25*. All PCR products were single amplicons and subsequently sequenced. Combining these with the sequences from GenBank and PubMed, a database of 89 full-length sequences of *Pfs25* of *P. falciparum* isolates in Thailand were generated (Additional file 2: Table S1).

Sequence analysis of *Pfs25* identified five additional haplotypes: H3 to H7. In total, there were seven *Pfs25* haplotypes in the *P. falciparum* population in Thailand (Fig. 1 and Additional file 3: Table S2). Most of the sequences were H2, representing 69% of the parasite population in Thailand, and H2 was also the most widely distributed haplotype as it was detected in all regions. H1 was the second major haplotype, representing 15% of the parasite population, and was highly prevalent in the northwestern regions of Thailand. The others were minor haplotypes and locality-specific. These included three haplotypes (H4, H5, and H7) that were specific to the *P. falciparum* populations in Yala, while H6 was detected in *P. falciparum* in Mae Hong Son and Kanchanaburi. More than one haplotypes co-existed in each parasite population, with an average haplotype diversity index (*Hd*) ± standard deviation (SD) of 0.507 ± 0.059. Interestingly, the highest diversity appeared in *P. falciparum* in Yala (southern Thailand), wherein four haplotypes were identified with *Hd* ± SD of 0.654 ± 0.106. The lowest diversity was detected in Trat (eastern Thailand), where two haplotypes were detected, with a *Hd* ± SD of 0.327 ± 0.153. This result indicates that the haplotype diversity of *Pfs25* could vary geographically in different parasite localities in Thailand.

Next, the distribution patterns of *Pfs25* haplotypes among the malaria parasite populations in Thailand were compared using Wright’s (*F*_*st*_) statistics. Table 1 shows that the *F*_*st*_ values were non-significant when the *Pfs25* haplotypes of *P. falciparum* from four provinces in the northwestern region (Mae Hong Son, Tak, Kanchanaburi, and Ranong) were compared. Non-significant *F*_*st*_ values were also detected between *P. falciparum* populations in eastern and southern Thailand (Ubol Ratchatani, Trat, and Yala). In contrast, significant *F*_*st*_ values were detected when the *Pfs25* alleles from the northwestern region were compared with those from the eastern and southern regions. This result indicates genetic differentiation in the *P. falciparum* populations in Thailand, wherein one comprised *P. falciparum* in northwestern Thailand and the other in eastern and southern Thailand.

**Table 1:** *F*_*st*_ analysis of *Pfs25* alleles in different populations of *Plasmodium falciparum* in Thailand

Origin	MH	K	TK	RN	TD	UB
K	0.01575 *P* = 0.28	0				
TK	0.40276 *P* = 0.02*	0.21298 *P* = 0.14	0			
RN	− 0.21980 *P* = 0.77	− 0.02995 *P* = 0.71	0.29766 *P* = 0.10	0		
TD	− 0.03086 *P* = 0.61	0.16062 *P* = 0.02*	0.64136 *P* = 0.00*	0.09518 *P* = 0.09	0	
UB	− 0.05186 *P* = 0.99	0.07249 *P* = 0.07	0.59351 *P* = 0.00*	0.01027 *P* = 0.30	− 0.03143 *P* = 0.58	0
YA	0.12240 *P* = 0.00*	0.18895 *P* = 0.01*	0.49092 *P* = 0.00*	0.17167 *P* = 0.00*	0.13916 *P* = 0.05	0.12500 *P* = 0.06

*MH* Mae Hong Son, *K* Kanchanaburi, *TK* Tak, *RN* Ranong, *TD* Trat, *UB* Ubol Ratchatani, *YA* Yala

^*^Statistically significant

### Global haplotype diversity of *Pfs25*

To compare the *Pfs25* haplotype diversity of *P. falciparum* in Thailand with other populations of *P. falciparum* worldwide, *Pfs25* sequences were retrieved from public databases. Our search identified 30 *Pfs25* sequences (10 full-length sequences and 20 partial sequences) in the “nucleotide collections” and 37 full-length *Pfs25* sequences in the “whole-genome shotgun” database in GenBank (https://www.ncbi.nlm.nih.gov/genbank/). Additionally, there were 218 sequences (202 full-length sequences and 16 partial sequences) in PlasmoDB (https://plasmodb.org/plasmo/app). Excluding the partial sequences as well as sequences of the same parasites or parasite with the same origins (i.e., 3D7 and NF54), there were a total of 218 full-length *Pfs25* sequences in the public databases. On combining these with the *Pfs25* sequences generated in this study, a global *Pfs25* database from 307 geographically different isolates of *P. falciparum* was established. The origins of the *P. falciparum* parasites in the global database were from 22 countries, including 10 countries in Africa (*n* = 173, including 3D7), 4 in Central and South America (*n* = 29), and 8 in Asia (*n* = 105; Additional File 2: Table S1).

**Fig. 2:**
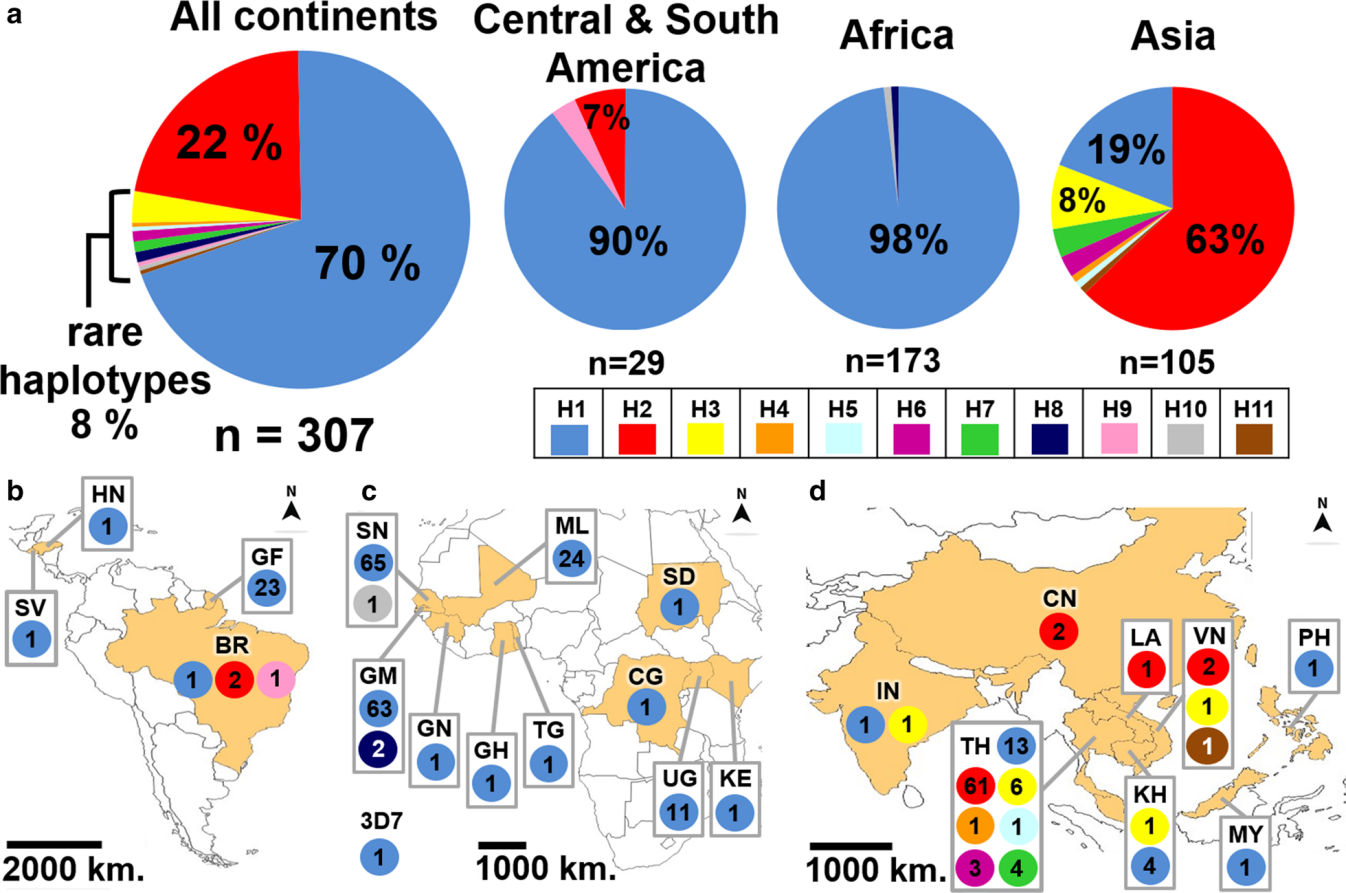
Global distribution of *Pfs25* haplotypes in *Plasmodium falciparum.* **a** The proportion of *Pfs25* in *P. falciparum* in all populations and in three continents. Numbers in the pie charts indicate the percentage of individual haplotypes. *n* indicates the number of samples. The distributions of *Pfs25* haplotypes in *P. falciparum* in **b** Central and South America, **c** Africa, and **d** Asia. Numbers in the circles in **b** to **d** indicate the number of parasites. Abbreviations: BR, Brazil; CG, Congo DR; CN, People's Republic of China; GF, French Guiana; GH, Ghana, GM, the Gambia; GN, Guinea; HN, Honduras; IN, India; KE, Kenya; KH, Cambodia; LA, Laos; ML, Mali; MY, Malaysia; PH, the Philippines; SD, Sudan; SN, Senegal; SV, El Salvador; TG, Togo; TH, Thailand; UG, Uganda; VN, Vietnam; 3D7, *Plasmodium falciparum* 3D7

The haplotype analysis of the *Pfs25* global database revealed 11 unique haplotypes. Of these, H1 and H2 were the dominant haplotypes, representing 70% and 22%, respectively, of the parasite populations (Fig. 2a). The other nine haplotypes were minor haplotypes, constituting 8% of the parasite population. The levels of *Pfs25* haplotype diversity were different in *P. falciparum* from different continents. For instance, *P. falciparum* from Central and South America had only three *Pfs25* haplotypes (*Hd* ± SD = 0.197 ± 0.095). Of these, H1 was detected in all countries, with a frequency of 90%, while H2 and H9 were detected in two and one *P. falciparum* isolates, respectively, in Brazil (Fig. 2b). *Plasmodium falciparum* from Africa also carried three haplotypes, H1, H8, and H10 (*Hd* ± SD = 0.034 ± 0.019). H1 was detected in all countries in Africa with a 98% frequency; meanwhile, H8 and H10 were rare haplotypes that were identified in two and one *P. falciparum* isolates in Gambia and Senegal, respectively, (Fig. 2c).

Interestingly, eight haplotypes were identified in *P. falciparum* in Asia (*Hd* ± SD = 0.564 ± 0.049), where H1 and H2 were the two major haplotypes, representing 19% and 63% of the parasite population in Asia. In addition to Thailand, H2 was also detected in Laos, People’s Republic of China, and Vietnam, whereas H1 was detected in Cambodia, Malaysia, the Philippines, and India (Fig. 2d). This result indicates that the highest *Pfs25* diversity in *P. falciparum* was detected in Asia. Additionally, the distribution patterns of *Pfs25* haplotypes in different parasite populations varied geographically, with H1 being the major haplotype in *P. falciparum* in Africa and South America and H2 in Asia.

To compare the *Pfs25* haplotype patterns among *P. falciparum* populations in three continents, Wright’s statistics were calculated between pairs of parasite populations. Significant *F*_*st*_ values were detected between all parasite pairs, being 0.67745 (*P* < 0.0001), 0.44847 (*P* < 0.0001), and 0.7858 (*P* = 0.00901) between *P. falciparum* in Asia and Africa, Asia, and South America, and Africa and South America, respectively. This finding indicated that population differentiation exists among *P. falciparum* populations in Asia, Africa, and South America, because unique haplotypes existed among different populations. As shown in Fig. 2, H8 and H10 were only detected in *P. falciparum* in Gambia and Senegal, H9 only in Brazil, and five other haplotypes (H4, H5, H6, H7, and H11) in Thailand and Vietnam.

### Haplotype network of *Pfs25*

**Fig. 3:**
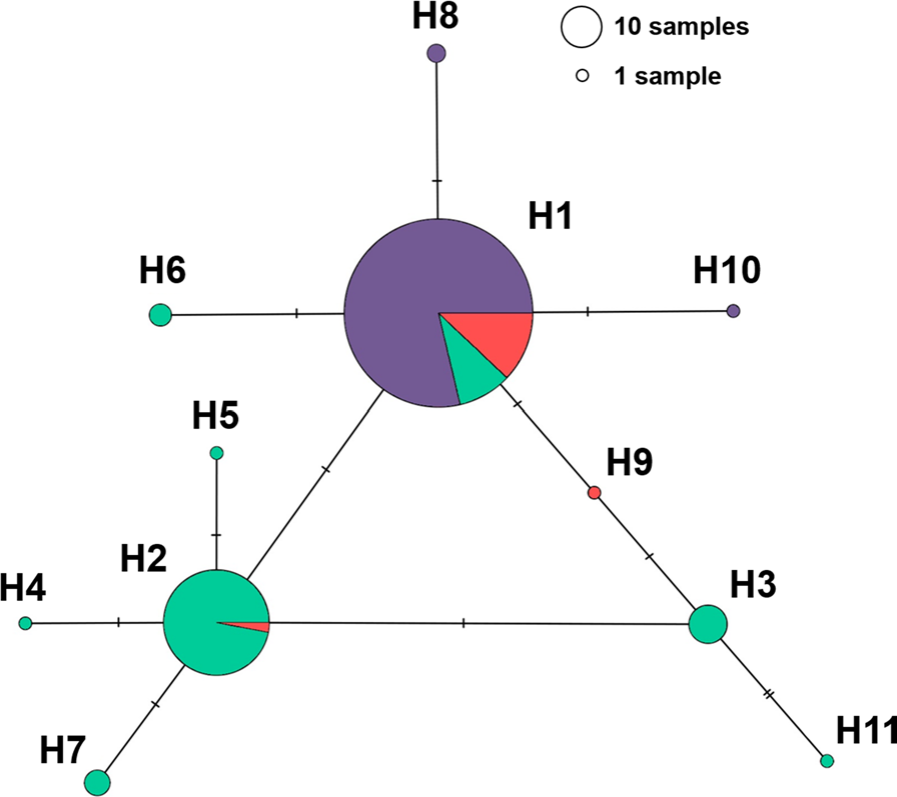
Haplotype network of *Pfs25* gene of *Plasmodium falciparum*. Each circle indicates a unique haplotype, and the sizes of the circles are proportional to the haplotype frequencies of each network. Each cross line represents one mutation step. Colors inside the circles indicate the origins of the *P. falciparum* isolates. Purple, Africa; green, Asia; red, Central and South America

To postulate the origins of *Pfs25* haplotypes, a *Pfs25* haplotype network was constructed. As shown in Fig. 3, the majority of rare *Pfs25* haplotypes were associated with one of the two major haplotypes, H1 and H2. Four minor haplotypes (H6, H8, H9, and H10) were closely associated with H1, while four other haplotypes (H3, H4, H5, and H7) were closely related to H2. Interestingly, H11, which was present in a single parasite isolate in Vietnam, was associated with H3. This result suggested that *Pfs25* minor haplotypes in *P. falciparum* from Africa, South America, and Central America could arise from H1, whereas *Pfs25* minor haplotypes from Asia could arise from H2.

**Fig. 4:**
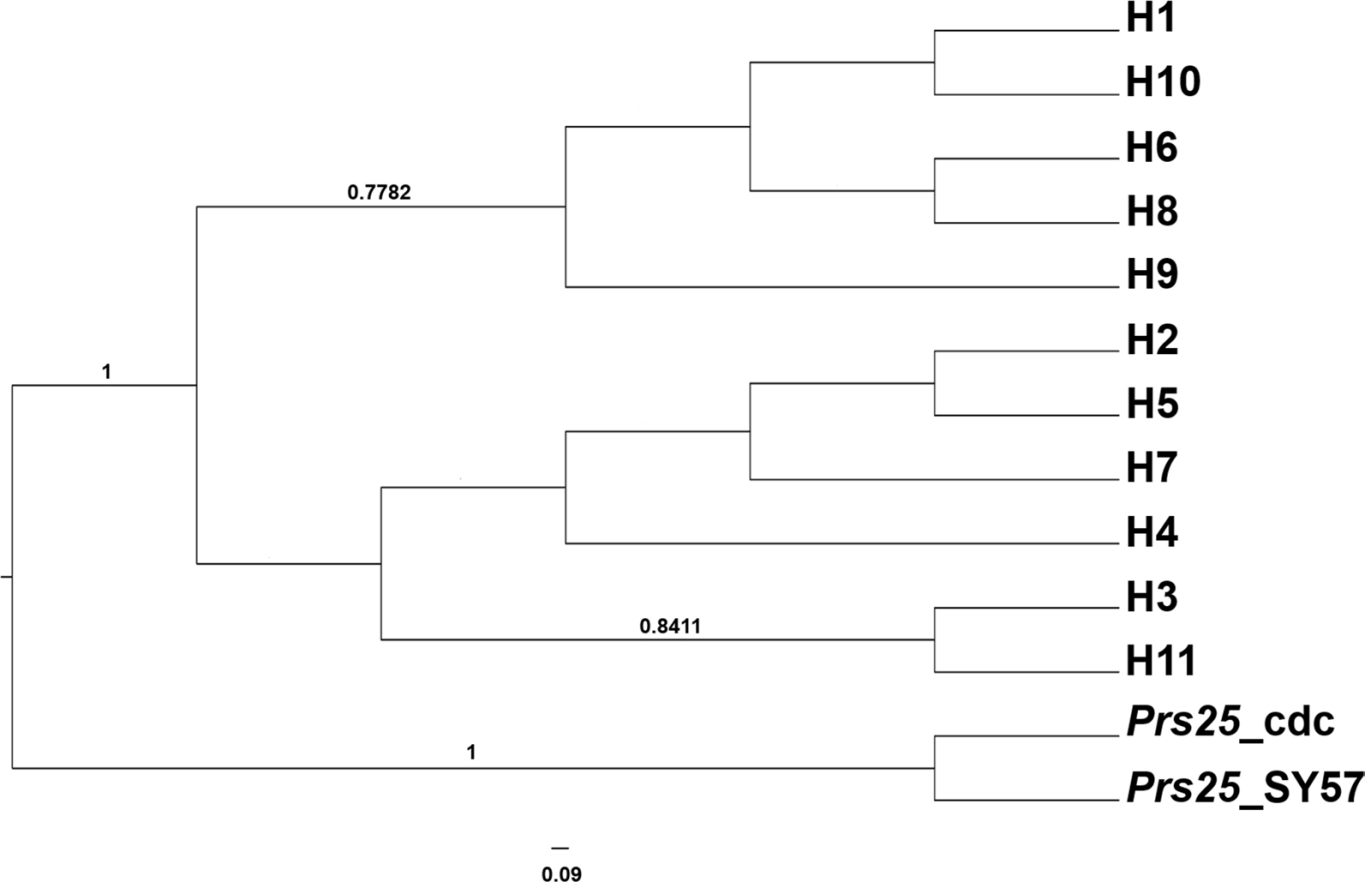
Bayesian inference phylogenetic tree (HKY + G model) of the *Pfs25* sequence of *Plasmodium falciparum*. Posterior probabilities > 0.5 are shown above the branches. Branch lengths are drawn to scale with the scale bar, representing 0.09 nucleotide substitution per site. Sequences of *Prs25*, the homolog gene of *Pfs25*, from *P. reichenowi* strains [57] were included

To further study the genetic relationship of *Pfs25* haplotypes, a BI phylogenetic tree was constructed. Consistent with the haplotype network, *Pfs25* haplotypes were clustered into three major clades (Fig. 4). The H1 clade was comprised of five haplotypes: H1, H6, H8, H9, and H10. The H2 clade was comprised of H4, H5, and H7, but two sub-clades were detected. H3 and H11 were separated into a distinct clade, although the H3 clade was more closely related to the H2 clade rather than the H1 clade. Unfortunately, the NJ tree was multifurcated and so excluded from analysis (Additional file 4: Fig. S2). Taken together, the results from the haplotype network and the phylogenetic tree consistently support that H1 and H2 could mainly contribute to the genetic diversity of *Pfs25* in *P. falciparum* populations.

### Molecular basis of *Pfs25* variations

**Fig. 5:**
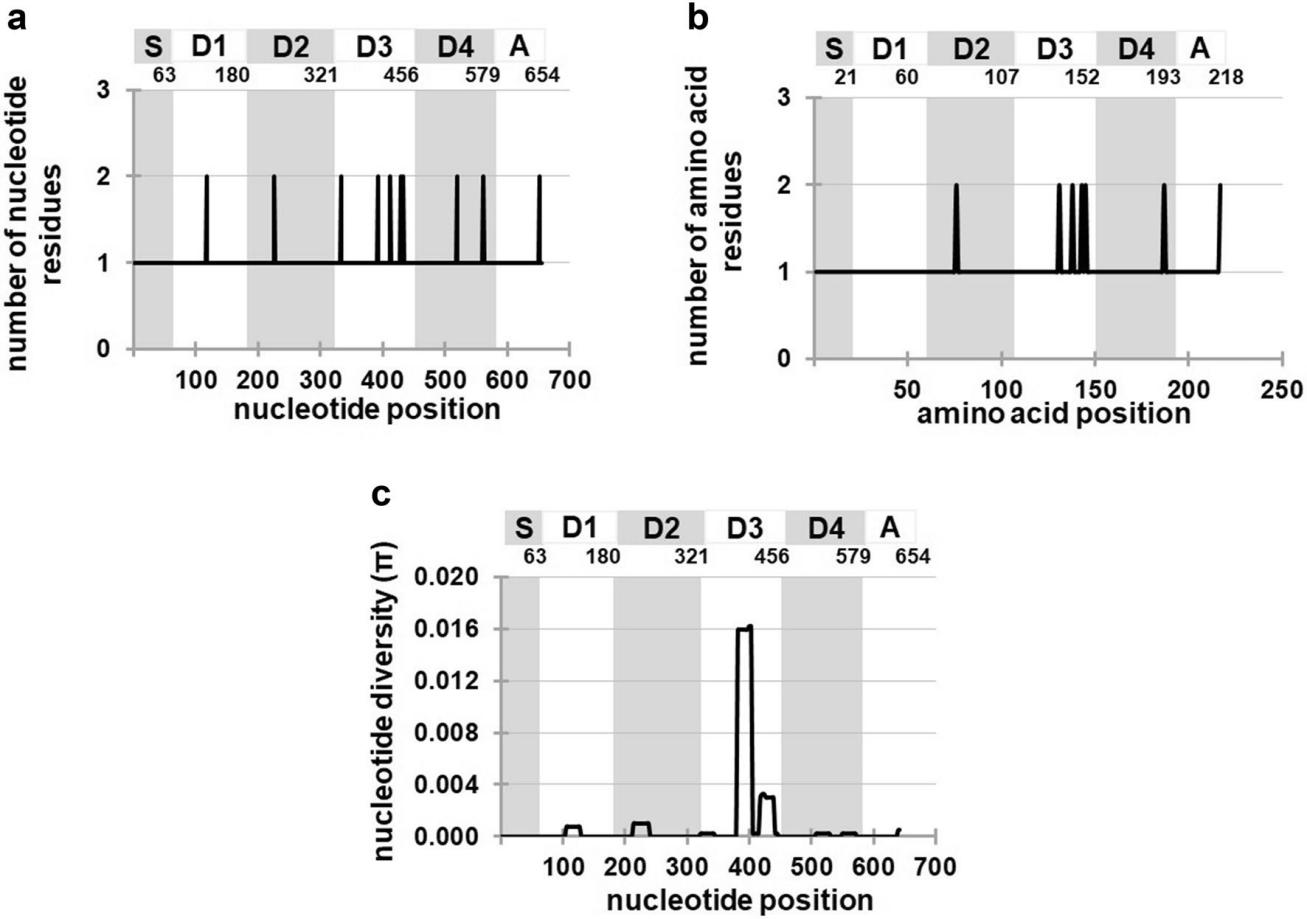
Nucleotide diversity in the *Pfs25* gene of *Plasmodium falciparum*. **a** Ten single-nucleotide polymorphism (SNP) sites in *Pfs25*. Each site contains two polymorphic nucleotides. **b** Seven amino acid substitution sites in Pfs25 antigen. **c** Sliding window of nucleotide diversity index (π). The π values were calculated using DnaSP version 6 with a window length of 25 bp and a step size of 3 bp. Bar shows regions corresponding to signal peptide (S), four EGF-like domains (D1 to D4), and transmembrane domain (A) in Pfs25

To investigate the sequence polymorphisms in *Pfs25*, nucleotide sequences of 11 unique haplotypes of *Pfs25* were aligned. The analysis identified ten single-nucleotide polymorphisms (SNPs) (Table 2 and Fig. 5a), with an average pairwise nucleotide difference between sequences (*k*) of 0.559 and an average nucleotide diversity at each locus (π) ± SD of 0.00085 ± 0.00007 (Additional File 5: Table S3). Of these, three SNPs at nucleotide positions 117 (AGT/AGC), 333 (AAG/AAA), and 519 (GGA/GGG) resulted in synonymous amino acid substitutions at amino acid residues 39 Ser, 111 Lys, and 173 Gly, respectively. The SNP frequencies at positions 117, 333, and 519 were T/C (0.992/0.001), A/G (0.997/0.003), and A/G (0.997/0.003), respectively. The other SNPs detected at the nucleotide positions 226 (GAT/AAT), 392 (GGA/GCA), 412 (GGC/AGC), 428 (GTA/GCA), 433 (GAT/AAT), 561 (AAT/AAA), and 651 (ATG/ATA) resulted in non-synonymous amino acid substitutions at amino acid residues 76 Asp/Asn, 131 Gly/Ala, 141 Gly/Ser, 143 Val/Ala, 145 Asp/Asn, 187 Asn/Lys, and 217 Met/Ile, respectively. The SNP frequencies at positions 226, 392, 412, 418, 433, 561, and 651 were G/A (0.987/0.013), G/C (0.726/0.274), G/A (0.997/0.003), T/C (0.964/0.036), G/A (0.997/0.003), T/A (0.997/0.003), and G/A (0.994/0.006), respectively.

**Table 2:** Single-nucleotide polymorphism (SNP) sites in the *Pfs25* gene of *Plasmodium falciparum*

Haplotype	Nucleotide position									
	117	226	333	392	412	428	433	519	561	651
H1	AGT	GAT	AAG	GGA	GGC	GTA	GAT	GGA	AAT	ATG
	Ser	Asp	Lys	Gly	Gly	Val	Asp	Gly	Asn	Met
H2	–	–	–	GCA	–	–	–	–	–	–
	–	–	–	Ala	–	–	–	–	–	–
H3	–	–	–	GCA	–	GCA	–	–	–	–
	–	–	–	Ala	–	Ala	–	–	–	–
H4	–	–	–	GCA	–	–	AAT	–	–	–
	–	–	–	Ala	–	–	Asn	–	–	–
H5	–	–	AAA	GCA	–	–	–	–	–	–
	–	–	Lys	Ala	–	–	–	–	–	–
H6	AGC	–	–	–	–	–	–	–	–	–
	Ser	–	–	–	–	–	–	–	–	–
H7	–	AAT	–	GCA	–	–	––	–	–	–
	–	Asn	–	Ala	–	–	–	–	–	–
H8	–	–	–	–	–	–	–	–	–	ATA
	–	–	–	–	–	–	–	–	–	Ile
H9	–	–	–	–	–	GCA	–	–	–	–
	–	–	–	–	–	Ala	–	–	–	–
H10	–	–	–	–	–	–	–	GGG	–	–
	–	–	–	–	–	–	–	Gly	–	–
H11	–	–	–	GCA	AGC	GCA	–	–	AAA	–
	–	–	–	Ala	Ser	Ala	–	–	Lys	–
Frequency	0.990	0.987	0.997	0.726	0.997	0.964	0.997	0.997	0.997	0.994
of H1 SNP										

Underlined letters indicate SNP sites

The SNP frequency analysis showed that the frequencies of the H1 allele were higher than 0.95 at all SNP sites, except at position 392, where the frequency was 0.726. This indicated that most SNPs detected in *Pfs25* were rare. Notably, eight SNPs (positions 116, 226, 333, 412, 433, 519, 516, and 651) were haplotype-specific (Table 2). Interestingly, the SNP at nucleotide position 392 differentiated the two major haplotypes, H1 and H2. In the BI phylogenetic tree (Fig. 4), all *Pfs25* haplotypes (H6, H8, and H8) in the H1 clade carried 392 G, whereas all haplotypes in the H2 clade carried 392 C.

The SNP distribution analysis of *Pfs25* revealed that nine SNPs were located in the EGF domains and one SNP was mapped to the transmembrane (anchor) domain (Fig. 5b). Of these, five SNPs were mapped to EGF domain 3. No SNPs were observed in the signal peptide sequences. To further compare the levels of nucleotide diversity in different domains of *Pfs25*, a sliding window plot of the nucleotide diversity index (π) was analyzed (Fig. 5c). Results showed that the average π for EGF domains 1, 2, 3, and 4, and the transmembrane domain were 0.00017, 0.0018, 0.00361, 0.00011, and 0.00018, respectively. Thus, the sequence diversity was highest in the EGF domain 3 of *Pfs25*.

Additionally, the nucleotide diversity indices of *Pfs25* were calculated to compare the level of diversity among different malaria parasite populations. In agreement with the analysis of the haplotype diversity, the highest π and *k* values were detected in the *P. falciparum* population in Asia (Additional File 5: Table S3). Notably, π and *k* values of the *Pfs25* sequence from *P. falciparum* population in Asia were higher than the average values. Interestingly, although *P. falciparum* in Africa and South America were comprised of three haplotypes of *Pfs25*, the π value of *Pfs25* in *P. falciparum* in Africa was lower than that of South and Central America. When the levels of nucleotide diversity were analyzed for *P. falciparum* in individual countries (excluding countries with one sample), *Pfs25* in Asia (Thailand, Cambodia, India, and Vietnam) and Brazil had a nucleotide diversity higher than the average (Additional File 5: Table S3). In contrast, *Pfs25* from the parasite population from all African countries and in French Guiana exhibited an extremely low sequence diversity. No sequence variation was observed in *P. falciparum* in Mali (*n* = 24), Uganda (*n* = 11), and French Guiana (*n* = 23). This reveals that haplotype and nucleotide sequence diversity in *Pfs25* of *P. falciparum* in Asia is higher than that in the other parasite populations.

### Signature of natural selection against *Pfs25*

**Fig. 6:**
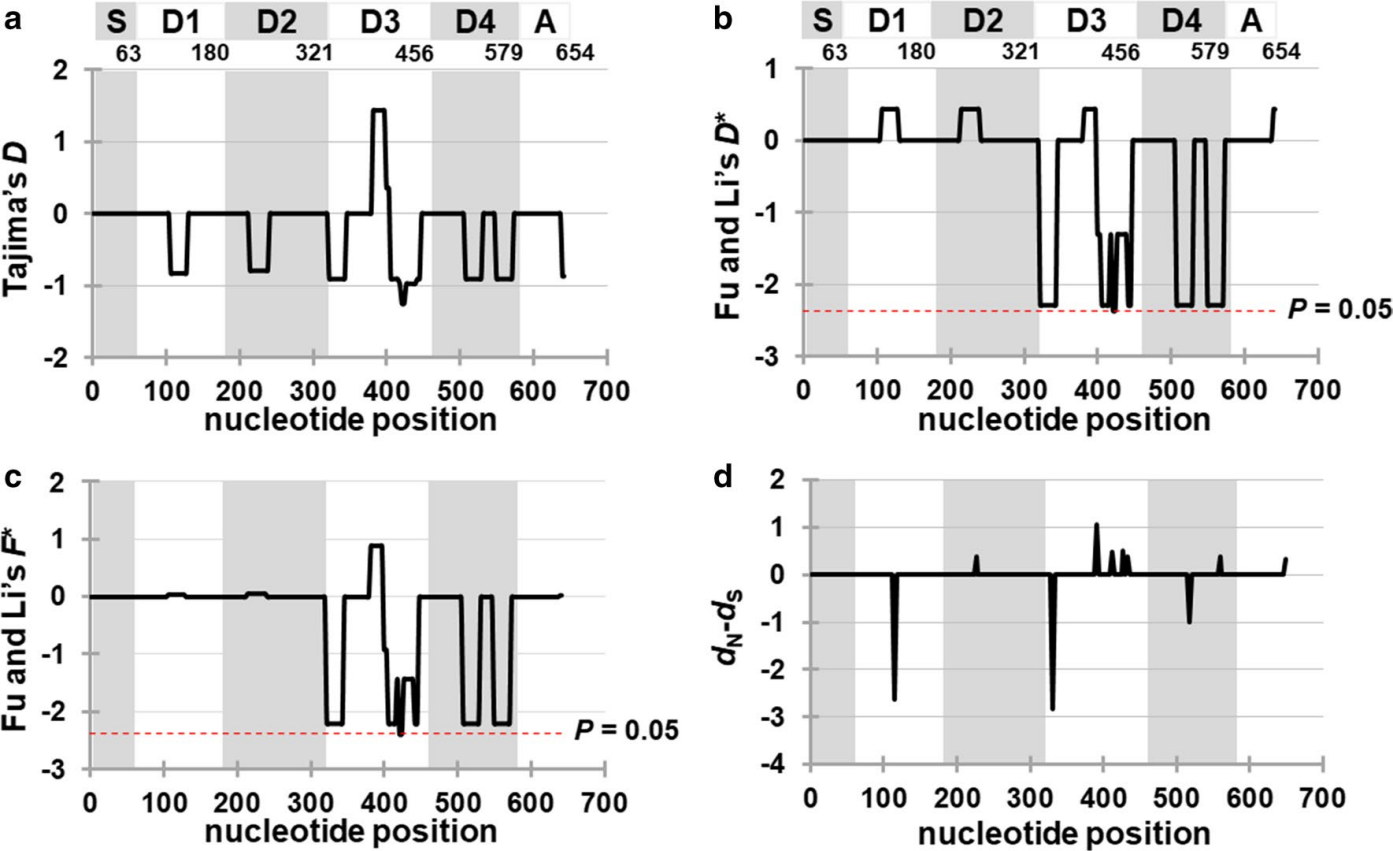
Sliding window plots of **a** Tajima’s *D* values, **b** Fu and Li’s D* values, **c** Fu and Li’s F* values, and **d** dN-dS values of the *Pfs25* gene of *Plasmodium falciparum*. The X axis shows the midpoint of the contiguous windows of 25 bp with a step size of 3 bp for the portions of the genes sequenced (nucleotide positions of each gene are given as those in the allele of the reference genome strain 3D7 [45]). Significant D* and F* values, representing the windows with significant departures from zero, were detected at the same nucleotide positions 409 to 436, respectively. The red lines indicate *P* values of < 0.05. Bar shows regions corresponding to signal peptides (S), four EGF-like domains (D1 to D4), and transmembrane domain (A) in Pfs25

To determine the evidence of natural selection on *Pfs25*, three neutrality tests were performed. Fu and Li’s D* and Fu and Li’s F* statistic tests were negative and significant (D* = − 2.78081, *P* < 0.05; F* = − 2.74867, *P* < 0.05), whereas Tajima’s *D* test was negative, but not significant (*D* = − 1.43957, *P* > 0.10; Additional File 5: Table S3). When the neutrality tests were analyzed using the sequence data from the parasite populations of each continent, non-significant values were detected in all tests. Furthermore, the sliding window plots of Tajima’s *D*, Fu and Li’s D*, and Fu and Li’s F* values were constructed. The evidence of negative selection was detected in EGF domain 3 of *Pfs25* in the sliding window plots of Fu and Li’s D* and Fu and Li F* tests (Fig. 6). Additionally, the *d*_*N*_˗*d*_*S*_ value of *Pfs25* was also analyzed using all parasite populations. The test showed a positive, but non-significant value (*z* test = 1.01, *P* = 0.31). When the *d*_*N*_˗*d*_*S*_ values were calculated using the sequence datasets, the results were positive and non-significant for the parasite populations in Asia and South America (Additional File 5: Table S3). Negative and non-significant *d*_*N*_˗*d*_*S*_ value was detected in the parasite populations in Africa. This result showed the tendency of negative selection operating in *Pfs25*.

### Contribution of the G131A mutation to the structural conformation of Pfs25

To predict the consequences of amino acid substitutions on the structure conformation of Pfs25, secondary structures of Pfs25 variants were predicted using JPred4 [81]. Analysis of the H1 variant identified coil structures at nine sites of amino acid residues (39, 76, 111, 138, 143, 145, 173, 187, and 217) and a beta-sheet structure at amino acid residue 131. The analyses of the ten other variants revealed the states of the secondary structures were similar to that of H1 (Additional file 6: Fig. S3), suggesting that the amino acid substitutions in Pfs25 are unlikely to affect the structural conformation of Pfs25.

**Fig. 7:**
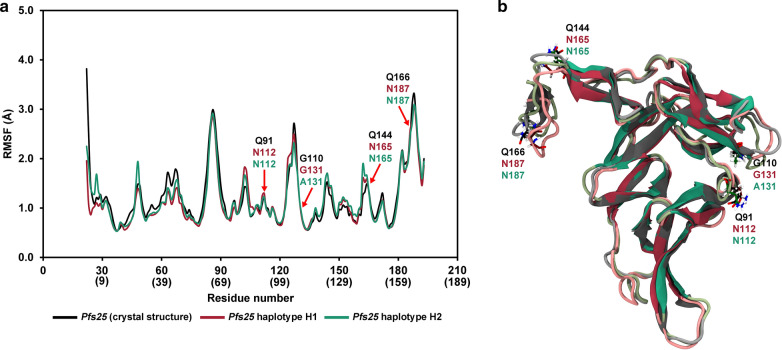
Predicted structures of the haplotypes 1 and 2 of *Pfs25* of *Plasmodium falciparum.* **a** Root mean square fluctuation (RMSF) plots of the crystal structure of *Pfs25* (black) and the homology models of *Pfs25* haplotypes H1 (pink) and H2 (green). **b** The representative structures, which were the structures closest to the average structures of the 80–100 ns molecular dynamics (MD) trajectories of the crystal structure of *Pfs25* (black) and the homology models of *Pfs25* haplotypes H1 (pink) and H2 (green)

Furthermore, MD was performed on the crystal structure of *Pfs25* (PDB ID: 6PHB [8]) and the homology models of *Pfs25* haplotypes H1 and H2 to gain insight into the effects of the G131A mutation of H2 on the structural conformation. This mutation was mapped to EGF-domain 3, which contains binding sites of transmission-blocking antibodies [82–84]. The crystal structure of *Pfs25* contains the unique amino acid residues of Q91, Q144, and Q166, whereas they are N112, N165, and N187 in H1 and H2. The MD results showed that the RMSF value trends of the crystal structure of *Pfs25* and homology models of H1 and H2 were relatively similar (Fig. 7a), indicating that the flexibilities of these three proteins are quite similar. The structure closest to the average structure of the 80–100-ns trajectory of each system was selected as the representative structure for the crystal structure of *Pfs25* and homology models of H1 and H2. The superimposition of the three representative structures showed that their overall structures were relatively similar (Fig. 7b). Specifically, the backbone RMSD values of the differences between the crystal structure and H1, crystal structure and H2, and H1 and H2 were 1.19 Å, 1.04 Å, and 1.17 Å, respectively. The percentages of secondary structures (helix, β-sheet, and random coil) of all three proteins (Table 3 and Additional file 7: Fig. S4) were also predicted to be relatively similar, suggesting that the structural conformations of H1, H2, and the crystal structure of *Pfs25* were relatively similar despite the fact that the crystal structure of *Pfs25* had three unique residues that were different from those in H1 and H2 and that H2 was different from H1 because of the G131A mutation.

**Table 3:** Percentages of β-sheet, helix, and random coil of the crystal structure of *Pfs25* and the homology models of *Pfs25* haplotypes H1 and H2 as calculated by defined secondary structure of protein (DSSP)

System	β-sheet (%)			Helix (%)				Random coil (%)			
	Parallel	Anti-parallel	Total	α- helix	3_10_-helix	π-helix	Total	Coil	Turn	Bend	Total
*Pfs25* (crystal structure)	1.2	38.1	39.3	0.0	2.5	0.0	2.5	26.5	21.6	10.1	58.2
*Pfs25* haplotype H1	1.2	38.9	40.1	0.0	2.2	0.0	2.2	26.9	19.1	11.7	57.7
*Pfs25* haplotype H2	1.3	38.3	39.6	0.0	2.5	0.0	2.5	26.9	19.3	11.7	57.9

Overall, these findings suggest that the polymorphisms in the major *Pfs25* haplotypes (G131A mutation) do not cause significant structural changes in terms of the secondary structures and stability. As *Pfs25* is essential to the malaria parasite, it should, therefore, be a potential target for a malaria transmission-blocking vaccine.

## Discussion

Here, a database containing 307 full-length *Pfs25* sequences of *P. falciparum* isolates worldwide was generated, revealing the extent of genetic diversity of *Pfs25* in different malaria parasite populations. To date, only three haplotypes of *Pfs25* sequences of *P. falciparum* are known (H1, H2, and H3). The first sequence was obtained from *P. falciparum* 3D7 (clone of NF54) [12]. Then, in an analysis of *P. falciparum Pfs25* sequences from a small number of isolates from Thailand [10], one SNP at nucleotide position 392 (GGA/GCA) was identified. This SNP classified *Pfs25* into two haplotypes: H1 (392G) and H2 (392C). Subsequently, in a partial sequence analysis of *Pfs25* from India (*n* = 155) [42, 43], another SNP was reported at nucleotide position 428 (GTA/GCA), resulting in the identification of a novel *Pfs25* haplotype, named H3. Thus, the three haplotypes constitute: H1 (392G and 428 T), H2 (392C and 428 T), and H3 (392C and 428C). Since then, there has been no systematic sequence analysis of *Pfs25* in the global *P. falciparum* population. Here, 224 sequences deposited in public databases were retrieved and combined with the *Pfs25* sequences of 83 isolates of *P. falciparum* from malaria hotspots in different regions of Thailand. Our sequence analysis revealed 11 haplotypes, of which 8 (H4–H11) were novel.

Of the 11 haplotypes identified, H1 and H2 represented 70% and 22% of the parasite populations, respectively, and so were considered the major *Pfs25* haplotypes. H1 was the most widely distributed haplotype in *P. falciparum* populations in three continents (Asia, Africa, and America), whereas H2 haplotype was distributed mainly in Asia (including Thailand, China, Laos, and Vietnam) and Brazil, but was absent in *P. falciparum* in Africa. Haplotype frequency analysis revealed that H1 accounted for approximately 90% of the *P. falciparum* populations in Africa and South America, while H1 and H2 had 19% and 63% frequencies, respectively, in *P. falciparum* in Asia. This result indicated that different major haplotypes of *Pfs25* represented different malaria geographical locations.

Our study also revealed the different levels of genetic diversity of *Pfs25* between *P. falciparum* populations in Asia, Africa, and South America. The highest diversity was detected in *P. falciparum* in Asia, where eight haplotypes were reported. This included the two major haplotypes (H1 and H2) and six minor haplotypes (H3–H7 and H11). Seven haplotypes were detected in *P. falciparum* in Thailand, while in other *P. falciparum* populations only one or two haplotypes were identified. Interestingly, haplotypes H1, H2, and H3 could be detected in China and many countries in Southeast Asia. Moreover, these three haplotypes are present in *P. falciparum* populations in India, indicating a close genetic relatedness and gene flow between these *P. falciparum* populations. This result was in agreement with our analysis of *MSP-3* sequences [40].

In addition, *P. falciparum* in Asia had the highest number of rare haplotypes. According to the haplotype network analysis, the minor haplotypes may have originated from different major haplotypes, where H4, H5, and H7 were most closely related to H2, whereas H6 was closely related to H1. Additionally, H11 and H3 were more closely related to each other than any other haplotypes. Although the number of haplotypes in *P. falciparum* in Asia was the highest, the real number of haplotypes in the malaria populations is likely to be underestimated since a large dataset (*n* < 50) was available from only the *P. falciparum* population in Thailand. Therefore, *P. falciparum* samples from other countries in Southeast Asia should be included in further studies to generate a complete view of the parasite diversity in Asia.

In contrast, a lower genetic diversity appeared in *P. falciparum* populations in Africa, even though the dataset comprised 171 *Pfs25* sequences. Of the three haplotypes identified, H1 was present in all countries in Africa and accounted for 98% of the populations. Additional novel haplotypes H8 and H10 were identified in *P. falciparum* in Gambia and Senegal. Based on the haplotype network, these haplotypes were closely related to H1, indicating that the origins of *Pfs25* diversity of *P. falciparum* in Africa were mainly from H1. A low genetic diversity of *Pfs25* was also detected in *P. falciparum* populations in South America. Of the three haplotypes identified, H1 was detected in all countries in South America, while H2, the major haplotype in Asia, was detected in two isolates in Brazil. This result implies that H2 could be due to a single mutation from H1, rather than the introduction of the parasites from Asia. In accord, H9 was also a minor haplotype in Brazil, and according to the haplotype network analysis, H9 could have arisen from the H1 haplotype. Taken together, the haplotype network analysis demonstrated the extent of genetic diversity of *Pfs25* in endemic malaria populations, which could have originated from the mutations of the two major haplotypes, H1 and H2.

Our data showed that *P. falciparum* populations in different endemic regions had unique *Pfs25* haplotype distribution patterns. This was supported by Wright’s *F*_*st*_ statistics, which indicated population subdivision among the *P. falciparum* populations in Asia, Africa, and South America. This finding was consistent with our previous analyses, wherein *AMA-1* and *MSP-3* sequences of *P. falciparum* from different endemic populations were sequenced [39, 40]. In these studies, significant *F*_*st*_ values were observed between *P. falciparum* in Thailand and countries in Africa and South America. Similarly, in an analysis of *Pfs48/45* sequences of *P. falciparum* in western Kenya, Thailand, and Venezuela [85], a strong population subdivision existed between the parasites of different continents. Genetic isolation among these malaria parasite populations is likely because of strong constraints in gene flow. In contrast, our study showed that H3, a minor haplotype, was prevalent in many parasite populations in Asia, including Thailand, Cambodia, Vietnam, and India. Similarly, in our previous study, shared rare haplotypes of *AMA-1, MSP-3*, and *GLURP* have also been detected in *P. falciparum* populations from Thailand, Myanmar, and India [38–40]. Taken together, these results reveal a high gene flow among the parasite populations in Southeast and South Asian countries.

Surprisingly, the genetic diversity of *Pfs25* in *P. falciparum* in Asia was higher than that in Africa. This was in contrast to many genetic studies that have shown that the transmission intensity of the malaria parasite is highest in *P. falciparum* in Africa, which contributes to the extensive polymorphism of the parasites [86–88]. Recently, genome sequence analysis using Illumina Sequencing Technology was conducted to explore the nucleotide variations in the genomes of 7113 *P. falciparum* isolates worldwide [89]. According to the MalariaGen database (https://wellcomeopenresearch.org/articles/6-42/v1), *P. falciparum* isolates in Africa are genetically more diverse than those in Asia. In the MalariaGen, additional 31 novel SNPs were identified, which were not reported in the present study (Additional file 8: Table S4a). These findings were, however, not unexpected since the numbers of *P. falciparum* isolates in our database were much smaller than those in the MalariaGen. Almost all these SNPs were region-specific and could only be detected in very small numbers (< 10 isolates per site) of *P. falciparum* isolates. The origins of the isolates with the novel SNPs included Benin, Cameroon, Congo DR, Ghana, Guinea, Kenya, Mali, Malawi, and Tanzania in Africa. Novel rare region-specific SNPs were also found in *P. falciparum* from Thailand, Bangladesh, and Papua New Guinea. However, it should be noted that two-thirds of the SNPs were synonymous and so were unlikely to result in novel haplotypes. According to the SNP data in the MalariaGen [89], the genetic diversity in *Pfs25* of *P. falciparum* in Africa could be underestimated or much higher than in Asia. Thus, further investigation should be continued in these parasite populations to obtain a more complete global view of *Pfs25* diversity.

In addition, the number of parasites with at least one SNP (or the parasites with non-H1 haplotype) in the MalariaGen could also be identified (Additional file 8: Table S4b). Of the 7,113 samples in the MalariaGen database, sequence polymorphisms in *Pfs25* were detected in 2,677 parasites, so the ratio of H1: non-H1 haplotypes was approximately 2:1 (62%: 37%) (Additional file 8: Table S4c). Of these, 2,555 parasites (80% of the parasites in Asia) with non-H1 haplotypes were identified in Asia, while only 106 (3% of the parasites in Africa) and 6 (15% of the parasites in South America) parasites with non-H1 haplotypes were identified in Africa and South America, respectively. Consistent with the present study, H1 was the major haplotype of *Pfs25* in all *P. falciparum* parasite populations, except in Asia.

High levels of antigenic polymorphisms may be the result of positive selection acting to maintain the genetic diversity and allow the parasite to escape the adaptive immune responses of the host [90]. However, on analyzing *Pfs25*, there was no evidence of positive selection, as revealed by three neutrality tests (Tajima’s *D* test, Fu and Li’s D*, and Fu and Li’s F*) and analysis of *dN˗dS* values. The lack of positive selection pressure in Pfs25 was because the *Pfs25* gene is mainly expressed in the post-fertilization stages (mainly in zygote and ookinetes) [12]. Possibly, the genetic diversity of the sexual stage antigens is driven by factors of the mosquito vectors. Molina-Cruz et al. (2020) have shown that parasites in different continents express different variants of the surface protein Pf47 [91]. The Pfs47 protein serves as “the key” that interacts with the receptor P47Rec (“the lock”) in the midgut of the mosquito, allowing it to evade the immune system of the mosquito. Pfs25 antigen interacts with laminin, a protein expressed on the surface of the epithelium in the midgut of the mosquitos [18]. It would be interesting to study the diversity of the laminin-coding gene (*LANB2*) in different species of *Anopheles* mosquitoes. Currently, there is one study showing that the laminin sequences of *A. gambiae* is highly conserved [92]. This could be beneficial to the transmission of *P. falciparum* isolates in Africa where the majority of the isolates carried the H1 haplotype. Interestingly, the global distribution map of the *Anopheles* vector shows that the species diversity of *Anopheles* in Asia is greater than in Africa and South America [93]. Therefore, future studies should focus on investigating whether the different mosquito species exhibit sequence diversity in *LANB2* and/or whether Pfs25 haplotypes are associated with the distributions of species of the malaria vectors. Understanding the extent of genetic diversity of the mosquito receptors in other continents would reveal newer insight into the evolution of antigenic genes expressing the sexual stages of the malaria parasites.

The nucleotide sequence alignment showed that the overall genetic polymorphism of *Pfs25* was low. Similarly, there are relatively few genetic polymorphisms in *Pfs28* of *P. falciparum.* In the gamete surface protein Pfs28, two polymorphic sites (72 Lys/Arg and 104 Asp/Ala) have been reported [94]. This could imply that these genes could be essential in the survival of the parasites [22]. Likewise, a low sequence diversity was observed in genes encoding antigens of the sexual stages, including *Pfs48/45* and *Pfs47* [85, 95]. The nucleotide diversity indices of *Pfs48/45* and *Pfs47* were similar to those of *Pfs25*. In contrast, genes expressing surface antigens of the blood stages exhibited extensive sequence polymorphism [38, 39]. This is likely to be because the numbers of blood stage malaria parasites are larger than the gametocytes in the host blood circulation and antigens of the blood stages could be exposed to host immunity for a longer period, thereby generating a stronger immune selection pressure. Therefore, the sexual stage antigens, including Pfs25, could potentially serve as candidates for transmission-blocking vaccines to fight against the antigenically diverse parasites [96].

The SNP distribution analysis revealed that, of the ten SNPs detected in the present study, nine were detected in all four EGF-like domains and one in the anchor domain of Pfs25. Interestingly, most non-synonymous SNPs were mapped to the EGF-domain 3, which has been implicated as a target of the transmission-blocking monoclonal antibodies, including 4B7, 1D2, and 32F81 [82, 83]. The SNP that defined the two major haplotypes, H1 and H2, was mapped to nucleotide 392 (GGA/GCA), which resulted in amino acid substitution at residue 131 (Gly/Ala). According to the MD results, no significant structural changes, in terms of the secondary structures (helix, beta-sheet, and random coil) and flexibility, were detected between H1 and H2. Sera from volunteers vaccinated with Pfs25 vaccine (derived from H1) could effectively block the transmission of parasites from Thailand and Burkin Faso and could be attributed to identical structures of major Pfs25 haplotypes from different parasite isolates [10]. Additionally, two non-synonymous SNPs sites at positions 226 and 561, resulting in the amino acid substitutions at positions 76 (Asp/Asn) and 187 (Asn/Lys), were mapped to EGF-domains 2 and 4 that also contained Pfs25-specific antibody binding sites [84]. According to the secondary structure predictions using the JPred 4 server, these two amino acid substitutions are unlikely to cause changes in the secondary structure of Pfs25. Given that the H1 and H2 haplotypes represent > 90% of the parasite populations and both variants were predicted to be structurally similar (based on JPred 4 and MD), these results suggest that current Pfs25 vaccines formulated using the *Pfs25* H1 haplotype (or sequences of 3D7 or NF54) should act effectively in all *P. falciparum* populations worldwide.

## Conclusions

The genetic diversity of *Pfs25* in different malaria parasite populations was low. H1 was the single major haplotype in *P. falciparum* in Africa and South and Central America, whereas H1 and H2 were the two dominant haplotypes circulating in *P. falciparum* in Asia. The low sequence diversity in natural populations of the malaria parasites could be due to negative selection, driven by the functional constraints of the protein. This presents a great opportunity for applying the existing Pfs25-based vaccines to control malaria worldwide. Understanding the sequence diversity of *Pfs25* could aid in monitoring the vaccine efficacy as well as developing an assay for detecting the malaria parasites in the mosquito stages to detect the prevalence of malaria transmission in the vectors.

## Supplementary Information

**Additional file 1: Figure S1.** Root mean square deviation (RMSD) plots of **a** the crystal structure of Pfs25, **b** Pfs25 haplotype H1, and **c** Pfs25 haplotype H2. The RMSD values of all atoms and backbone atoms are shown in black and gray, respectively.

**Additional file 2: Table S1.** List of *P. falciparum* isolates with full-length sequences of the *Pfs25* gene

**Additional file 3: Table S2.** Allelic distribution of the *Pfs25* gene in *Plasmodium falciparum* from Thailand. Abbreviations: *H*, the number of haplotypes; *Hd*, haplotype diversity index; SD, standard deviation.

**Additional file 4: Figure S2.** Neighbor-joining phylogenetic tree (HKY85 model) of the *Pfs25* sequence of *Plasmodium falciparum*. Bootstrap values (> 50%) are shown. Sequences of *Prs25*, the homolog gene of *Pfs25*, from *P. reichenowi* strains were used [57].

**Additional file 5: Table S3** Nucleotide diversity and signature of natural selection in *Pfs25* in the global population of *Plasmodium falciparum.* Abbreviations: *n,* number of samples, *H*, the number of haplotypes; *Hd*, haplotype diversity index; SNP, total number of single-nucleotide polymorphism sites; nsSNP, non-synonymous SNP; ssSNP, synonymous SNP; *k*, average pairwise nucleotide difference between sequences; *π*, average nucleotide diversity at each locus; nd, not determined because of insufficient sequence data.

**Additional file 6: Figure S3.** Secondary structure analysis of Pfs25 variants using JPred 4. H1 to H11 represent the variants of Pfs25 identified in the present study. Black and red asterisks are synonymous and non-synonymous SNPs, respectively. Blue vertical lines indicate the coil structure, mapped to amino acid positions 39, 76, 111, 138, 143, 145, 173, 187, and 217. Green vertical line indicates the β-structure at position 131. The topmost bar shows regions corresponding to signal peptides (S), four epidermal growth factor (EGF)-like domains (D1 to D4), and transmembrane domain (A) in Pfs25.

**Additional file 7: Figure S4.** Defined secondary structure of protein (DSSP) plots of **a** the crystal structure of *Pfs25*, **b** *Pfs25* haplotype H1, and **c** *Pfs25* haplotype H2.

**Additional file 8: Table S4. a** The list of SNPs in *Pfs25* from the MalariaGen database [89]. *SNPs detected the present study. Gray and blue boxes indicate SNPs specific to *P. falciparum* isolates in Africa and Asia regions. **b** The geographical distributions of the *P. falciparum* isolates with polymorphic SNPs in *Pfs25* in the MalariaGen database. **c** The estimated proportions of the H1 and non-H1 haplotypes of *Pfs25* in *P. falciparum* populations in three continents (Asia, Africa, and South America).

## Data Availability

Nucleotide sequences of *Pfs25* reported in this article are available in GenBank® database with the accession numbers: OK318571 to OK318653.

## Abbreviations

BI: Bayesian inference
DSSP: Defined secondary structure of protein
EGF: Epidermal growth factor
*H*: The number of haplotypes
*Hd*: Haplotype diversity index
MD: Molecular dynamics
*K*: Average pairwise nucleotide difference between sequences;*n,* number of samples
NJ: Neighbor-joining
PCR: Polymerase chain reaction
*π*: Average nucleotide diversity at each locus
RMSD: Root mean square deviation
RMSF: Root mean square fluctuation
SNP: Single-nucleotide polymorphism
SD: Standard deviation
SNP: Total number of single-nucleotide polymorphism sites
nsSNP: Non-synonymous SNP
ssSNP: Synonymous SNP
