# Supplementary figures and images for: Global diversity of the gene encoding the Pfs25 protein—a Plasmodium falciparum transmission-blocking vaccine candidate
Source: Parasit Vectors. 2021 Nov 8;14:571. doi: 10.1186/s13071-021-05078-6 (PMC8574928; doi:10.1186/s13071-021-05078-6)

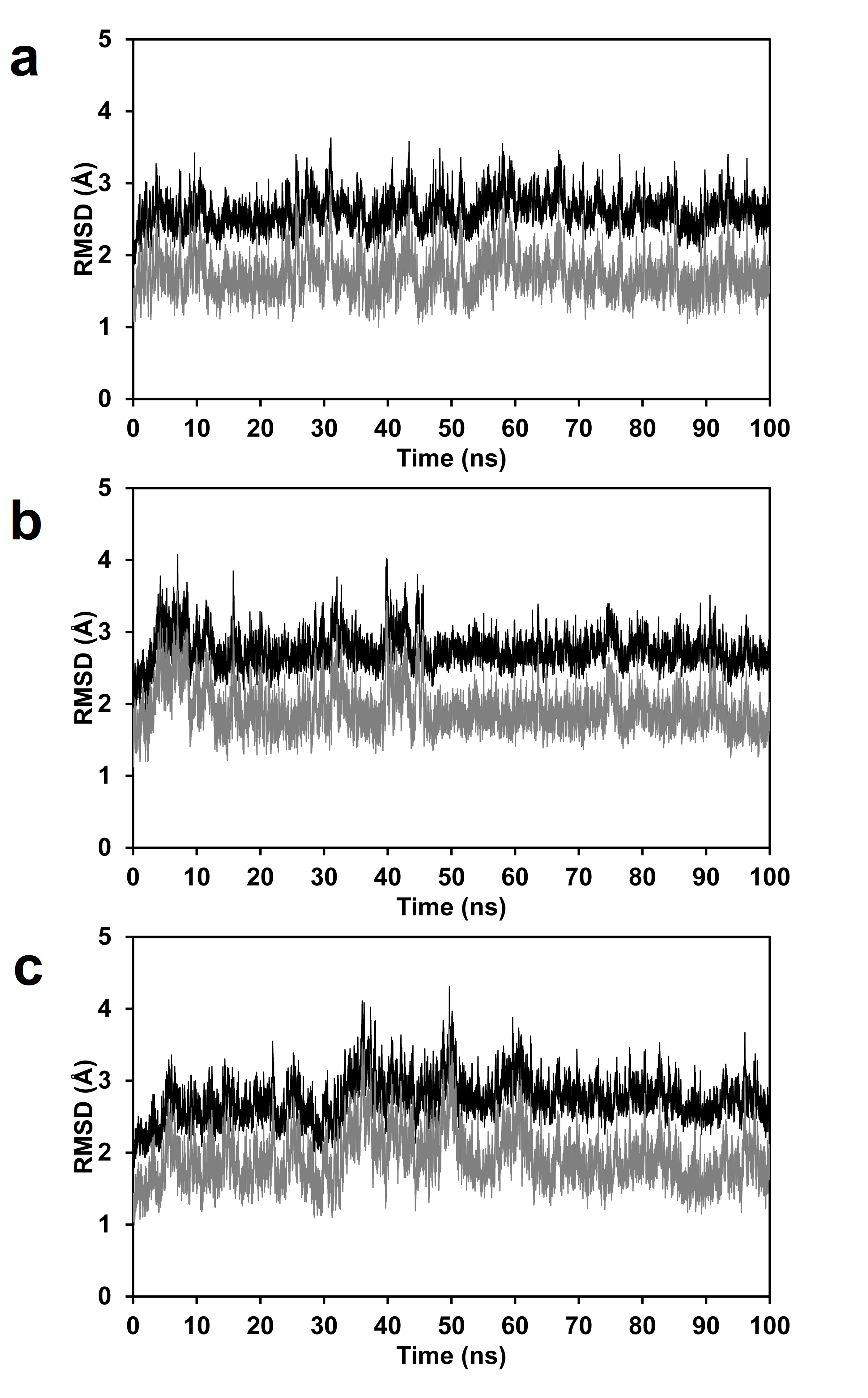

Supplement: Supplementary file 1 — Additional file 1: Figure S1. Root mean square deviation (RMSD) plots of a the crystal structure of Pfs25, b Pfs25 haplotype H1, and c Pfs25 haplotype H2. The RMSD values of all atoms and backbone atoms are shown in black and gray, respectively. [file 13071_2021_5078_MOESM1_ESM.tif]

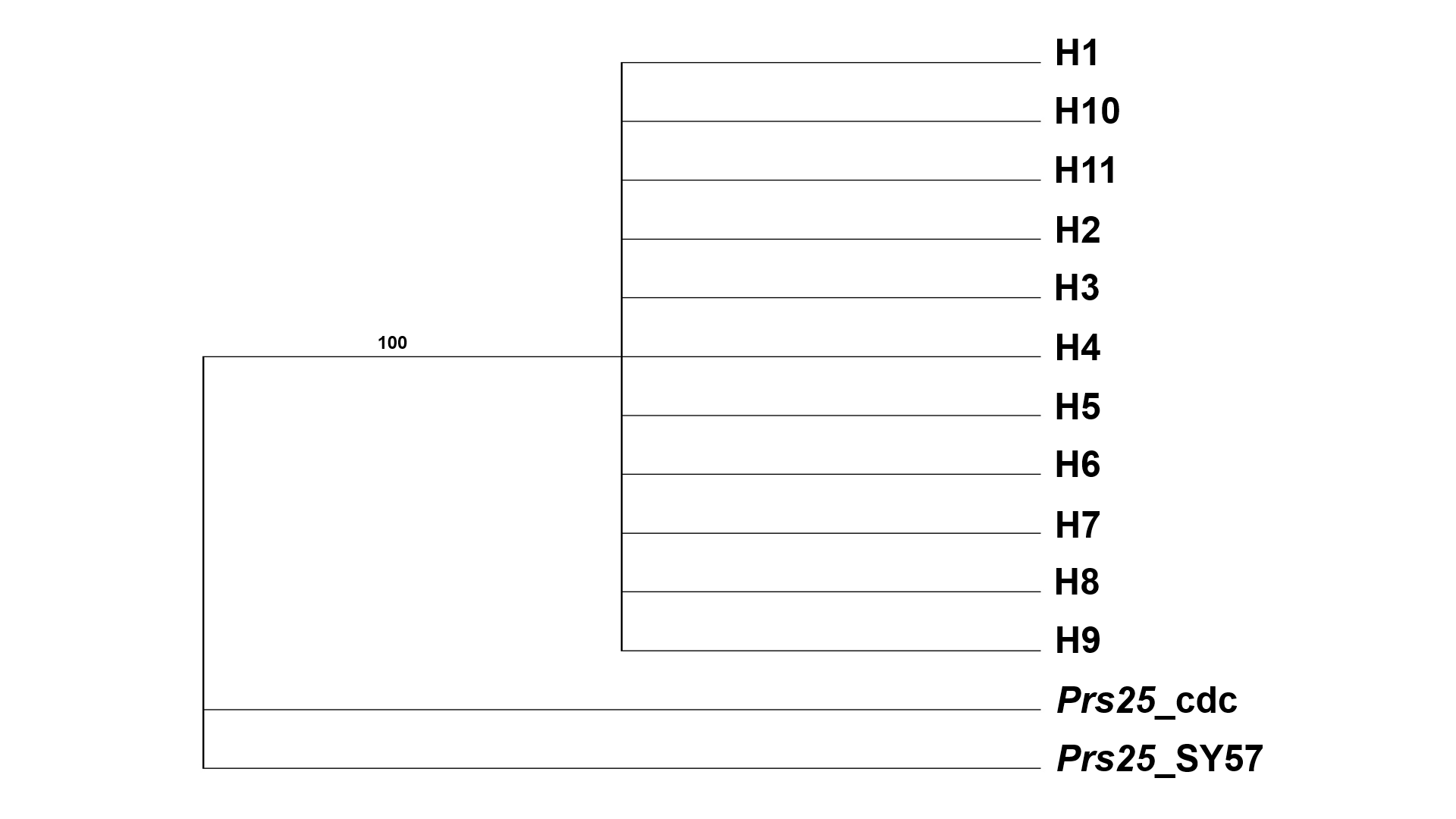

Supplement: Supplementary file 4 — Additional file 4: Figure S2. Neighbor-joining phylogenetic tree (HKY85 model) of the Pfs25 sequence of Plasmodium falciparum. Bootstrap values (> 50%) are shown. Sequences of Prs25, the homolog gene of Pfs25, from P. reichenowi strains were used [57]. [file 13071_2021_5078_MOESM4_ESM.tif]

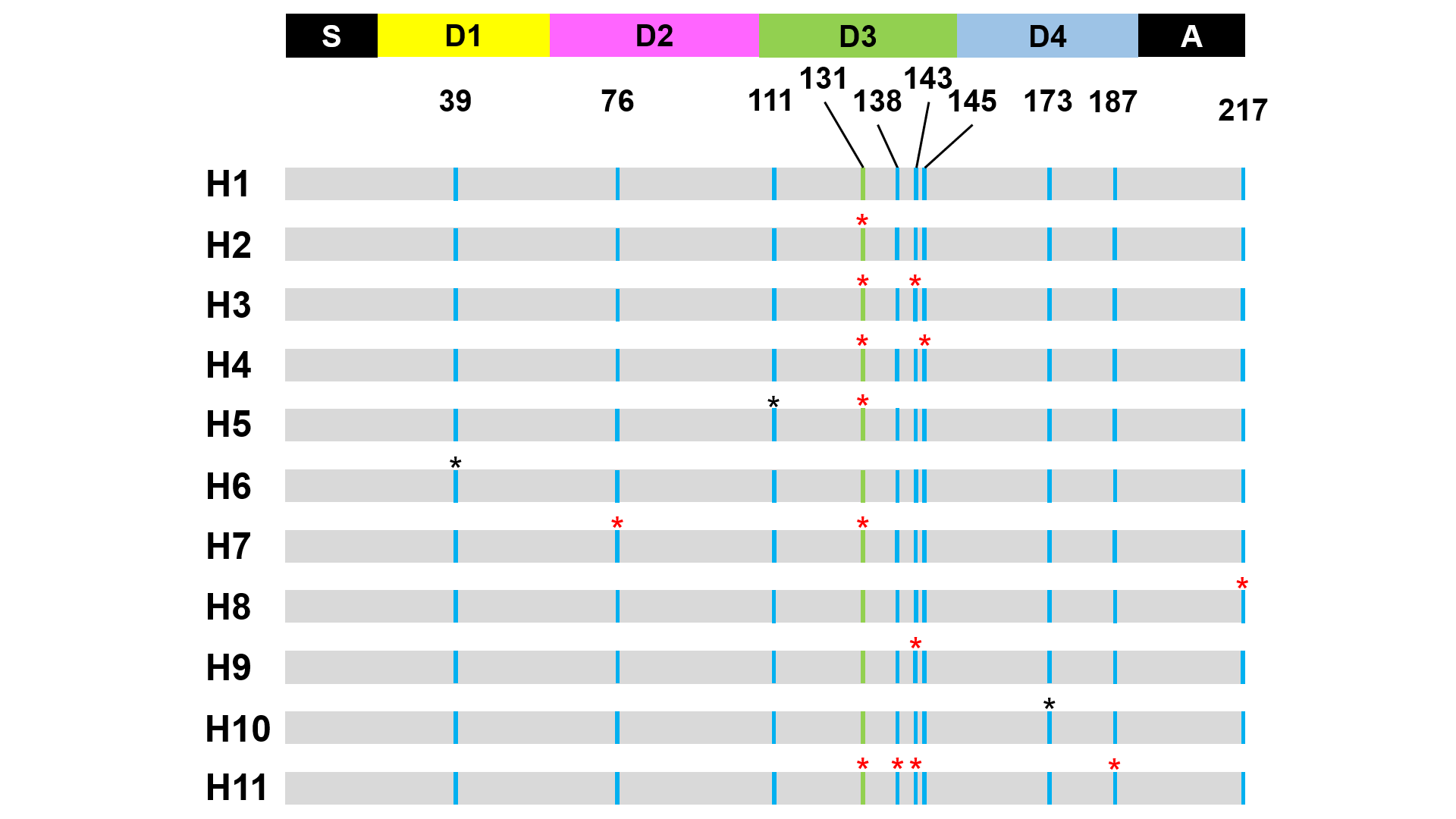

Supplement: Supplementary file 6 — Additional file 6: Figure S3. Secondary structure analysis of Pfs25 variants using JPred 4. H1 to H11 represent the variants of Pfs25 identified in the present study. Black and red asterisks are synonymous and non-synonymous SNPs, respectively. Blue vertical lines indicate the coil structure, mapped to amino acid positions 39, 76, 111, 138, 143, 145, 173, 187, and 217. Green vertical line indicates the β-structure at position 131. The topmost bar shows regions corresponding to signal peptides (S), four epidermal growth factor (EGF)-like domains (D1 to D4), and transmembrane domain (A) in Pfs25. [file 13071_2021_5078_MOESM6_ESM.tif]

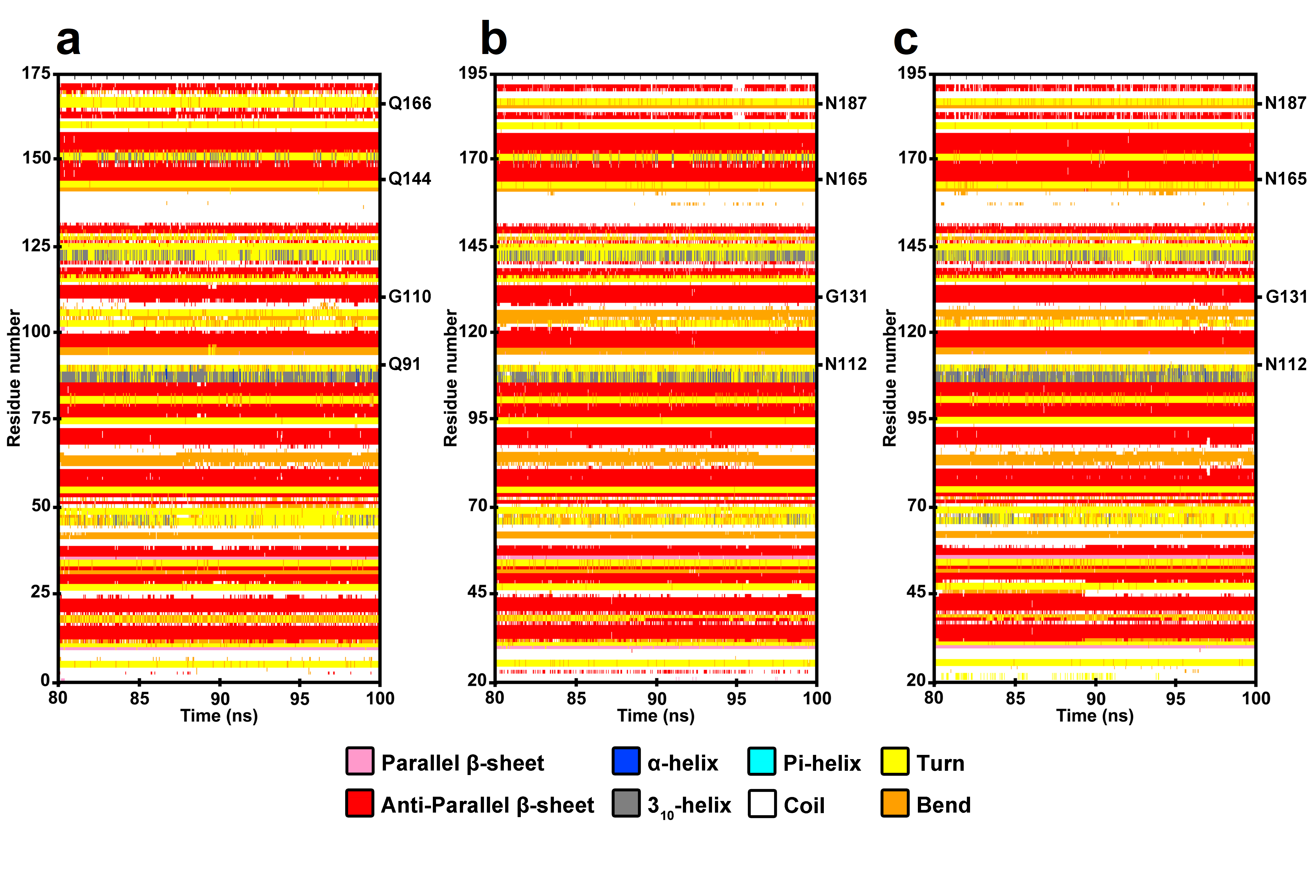

Supplement: Supplementary file 7 — Additional file 7: Figure S4. Defined secondary structure of protein (DSSP) plots of a the crystal structure of Pfs25, b Pfs25 haplotype H1, and c Pfs25 haplotype H2. [file 13071_2021_5078_MOESM7_ESM.tif]
